# Identification of laryngeal cancer prognostic biomarkers using an inflammatory gene-related, competitive endogenous RNA network

**DOI:** 10.18632/oncotarget.13627

**Published:** 2016-11-25

**Authors:** Qun He, Linli Tian, Hao Jiang, Jiarui Zhang, Qiang Li, Yanan Sun, Jiannan Zhao, Huijun Li, Ming Liu

**Affiliations:** ^1^ Department of Otolaryngology, Head and Neck Surgery, The Second Affiliated Hospital, Harbin Medical University, Harbin, China; ^2^ Department of Otolaryngology, Head and Neck Surgery, The First Affiliated Hospital, Harbin Medical University, Harbin, China; ^3^ The Affiliated Hongqi Hospital of Mudanjiang Medical University, Mudanjiang, China; ^4^ The Second Affiliated Hospital, Harbin Medical University, Harbin, China

**Keywords:** inflammatory gene, laryngeal cancer, microRNA, competetive endogenous RNA, prognostic biomarker

## Abstract

Competitive endogenous RNAs (ceRNAs) act as molecular sponges for microRNAs (miRNAs), and are associated with tumorigenesis in various cancers, including laryngeal cancer (LC). In this work, we constructed an LC-specific inflammatory gene-related ceRNA network (IceNet). In IceNet, ceRNAs targeting inflammation-related genes tended to be network hubs. Additionally, the betweenness centralities of these hub ceRNAs were higher than those of the inflammation-related genes themselves, indicating that the hub ceRNAs in this study played critical roles in communication between IceNet molecules. Gene Ontology and Kyoto Encyclopedia of Genes and Genomes pathway analyses indicated that IceNet molecules are associated with multiple cancer-related functions and signaling pathways. Using cFinder software and survival analyses, we identified a potential prognostic module within IceNet that contains 18 mRNAs and a long non-coding RNA (lncRNA), and we effectively stratified patients into high- and low-risk subgroups with different survival outcomes, independent of patient age and tumor grade. This 18-mRNA and one-lncRNA module provides a novel mechanism for potentially improving LC patient prognostic predictions. Applying the module clinically to differentiate high- and low-risk patients could inform therapeutic decision making and ultimately improve patient outcomes. In addition, these results demonstrate the potential importance of IceNet hub ceRNAs in LC development and progression.

## INTRODUCTION

Head and neck squamous cell carcinoma (HNSCC) is the sixth most common cancer in the world, with a mortality rate of ∼50%. Approximately 600,000 HNSCC cases occur annually [1], of which ∼25% are new laryngeal cancer (LC) cases [2]. Although surgical, radiotherapeutic, and chemotherapeutic technologies continue to improve, LC patient prognosis remains poor. Novel therapeutic targets are required to improve diagnosis and prognosis prediction, and ultimately survival outcomes, in LC patients.

As a critical component of tumor progression [3], cancer-related inflammation is now recognized as a cancer hallmark [4]. Recent efforts focused on the relationship between inflammatory cells and many types of cancers have revealed that inflammatory cells can occupy much of the tumor microenvironment, fostering tumor cell proliferation, survival and migration [3]. Signaling pathways that orchestrate innate inflammation, such as NF-kB, are activated in many cancers [5]. These findings provide insight into mechanisms of inflammation in promoting tumorigenesis. The present study focuses on novel LC prognostic biomarkers from the perspective of inflammation.

MicroRNAs (miRNAs) are small, non-coding RNAs (∼22 nucleotides long) that negatively regulate gene expression by targeting messenger RNAs (mRNAs). In cells, a pool of mRNAs, transcribed pseudogenes, long noncoding RNAs (lncRNA) [6], and circular RNAs (circRNA) [7, 8], together termed competitive endogenous RNAs (ceRNAs), competes for a complementary miRNA [9]. ceRNAs act as molecular sponges for miRNAs, targeting specific miRNA response elements (MREs) and ultimately de-repressing the miRNA target genes. ceRNAs have been linked to cancer through various oncogenes and tumor suppressors; for example, the pseudogene PTENP1 appears to compete with the important tumor suppressor gene PTEN for interaction with a miRNA, thus regulating PTEN protein levels [10].

In this study, we used a computational approach to construct an inflammatory genes-associated ceRNA network (IceNet) specific to LC. 109 LC patient gene expression profiles were obtained from the Gene Expression Omnibus (GEO) repository [11]. An LC-specific ceRNA network consisting of lncRNAs and mRNAs derived from expression data, along with experimentally validated miRNA-mRNA and miRNA-lncRNA interaction data, was constructed. To explore the roles of inflammatory genes and their ceRNAs in LC initiation and progression, we further constructed an LC-specific inflammatory gene-associated ceRNA network (IceNet). The results indicated that ceRNAs of known inflammatory genes tended to be network hubs. Additionally, the betweenness centralities (BC) of these hub ceRNAs were higher than those of the inflammatory genes themselves. Based on cFinder and survival analysis, we identified a potential prognostic module that contained 18 mRNAs and one lncRNA, H19, which is associated with tumor growth [12]. These analyses demonstrated that inflammatory gene-associated ceRNA networks might be used to identify novel laryngeal cancer-specific molecular biomarkers.

## RESULTS

### Analysis of IceNet topological properties and functional enrichment in LC

**Figure 1:**
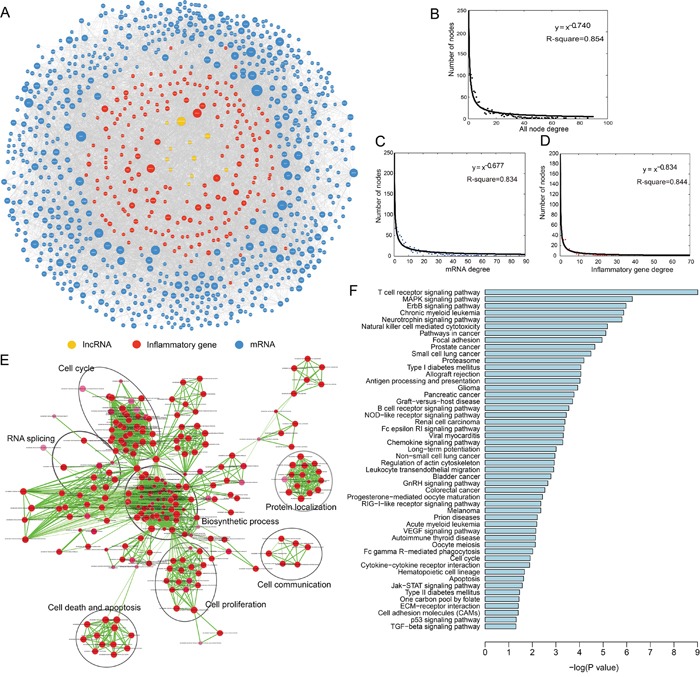
Topological properties of the inflammatory gene-associated ceRNA network (IceNet) **A**. The overview of IceNet. **B-D**. The degree distribution of all nodes, mRNA, and inflammatory genes in IceNet. Colors were assigned as the colors of the nodes in IceNet. **E**. The functional enrichment map of GO terms. Each node represented a GO term, which was grouped and annotated by GO similarity. Each edge represented whether there was any shared gene between two GO terms. Node size represented the number of genes in GO term. Color intensity of nodes was proportional to enrichment significance. Edge thickness represented the number of shared genes between two GO terms. Edge length was automatically arranged so that higher similar gene-sets placed closer together. **F**. Significantly enriched KEGG pathways of mRNAs in IceNET.

We analyzed 109 LC patient mRNA and lncRNA expression profiles from the publicly available Gene Expression Omnibus (GEO) database, along with experimentally validated miRNA and lncRNA interaction networks, to identify functional miRNA-mediated ceRNAs. A total of 13,665 miRNA-mediated ceRNA interactions were identified, including 377 lncRNA-mRNA and 13,288 mRNA-mRNA pairs. These functional interactions were integrated to build an LC-specific ceRNA network. We then extracted an inflammatory gene-related LC ceRNA network, IceNet, which included 935 nodes (including 190 inflammatory genes, 10 lncRNAs, and 735 mRNAs) and 5415 edges (Figure 1A, Supplementary Table 1). To explore the topological properties of IceNet, degree distribution analysis was performed in all nodes, mRNAs, and inflammatory genes (Figure 1B–1D). We observed that the degree distribution of all IceNet nodes closely followed a power law distribution with R^2^=0.854 (Figure 1B). Most nodes had relatively few interactions with others, and only a small portion of nodes had a large number of interactions. The topology analysis suggested that IceNet had a small-world organization.

We also performed a functional enrichment analysis of all IceNet mRNAs (including inflammatory genes and others) based on Gene Ontology (GO) and Kyoto Encyclopedia of Genes and Genomes (KEGG) pathways. IceNet mRNAs were enriched in 122 GO terms (false discovery rate (FDR) <0.05) mainly in seven functional clusters, including biosynthetic process, cell communication, cell cycle, cell death and apoptosis, cell proliferation, protein localization process, and RNA splicing (Figure 1E), along with 50 KEGG pathways (P<0.05), including cancer, focal adhesion, and several signaling pathways (Figure 1F, Supplementary Table 2). All enriched signaling pathways, including MAPK, NOD-like receptor, Jak-STAT and p53 signaling, are known contributors to LC pathogenesis [13–16].

### Hub inflammatory gene-specific ceRNAs played critical communication roles in IceNet

**Figure 2:**
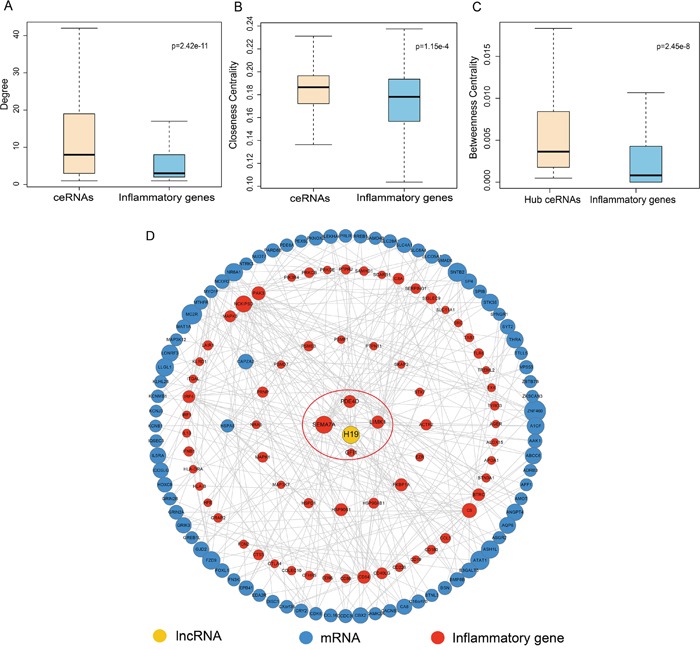
Comparison analysis of topological properties of ceRNAs and inflammatory genes in IceNet **A-B**. Box plot of degree and CC of ceRNAs and inflammatory genes in IceNet. **C**. Box plot of BC of hub ceRNAs and inflammatory genes in IceNet. **D**. Hub-subnetwork of IceNet.

We found that inflammatory genes had lower degree and closeness centralities (CC) than their ceRNAs in the IceNet (p=2.42e-11 for degrees, Figure 2A; p=1.15e-4 for CC, Figure 2B; Wilcoxon rank sum test), indicating that inflammatory gene ceRNAs were central within IceNet.

We next analyzed the hub inflammatory gene ceRNAs in IceNet in greater detail. In previous studies, hubs were typically defined as the top 10–20% of nodes in the networks by degree [17–19]. Thus, we chose the top 10% of nodes based on highest degree as the hub components, identifying 94 total hub nodes, including 11 inflammatory genes, and 83 ceRNAs (1 lncRNA and 82 mRNAs). The betweenness centralities of hub inflammatory gene ceRNAs were higher than those of the inflammatory genes themselves (p=2.45e-8, Figure 2C), indicating that these hub ceRNAs played critical roles in communicating with molecules in IceNet.

The lncRNA H19, in the IceNet hub-subnetwork (Figure 2D), reportedly promotes LC development and progression in combination with miR-148a-3p by competing with DNMT1 [20]. H19 interacted in IceNet with three hub inflammatory genes (LIMK1, PDE4D, SEMA7A). These observations demonstrated that hub inflammatory gene ceRNAs in IceNet were more likely to be essential for LC development and progression.

### Identification of inflammatory network-based prognosis-associated module biomarkers in LC

**Figure 3:**
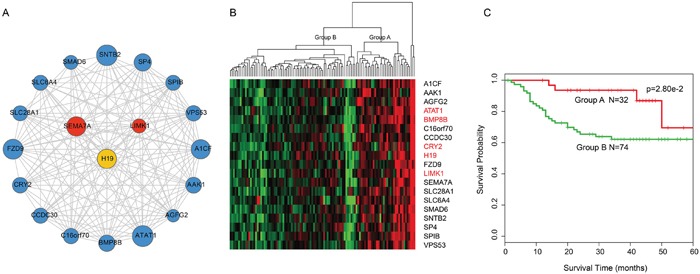
Overview of 18-mRNA and one-lncRNA module and its prognostic ability for assessing clinical outcome of laryngeal cancer **A**. Overview of the 18-mRNA and one-lncRNA module. **B**. Hierarchical clustering heatmap and dendrogram of patients based on the expression patterns of module molecules in laryngeal cancer. The genes and lncRNA in red represented cancer-associated genes and lncRNA. The genes in black represented the potential cancer-associated genes. **C**. KM survival curves of two subgroup patients resulted from the unsupervised hierarchical clustering in laryngeal cancer. P value was calculated using the log-rank test.

Based on cFinder software analysis, nine modules were identified in IceNet with k-clique>10 (Supplementary Table 3). We evaluated each module's ability to predict LC patient survival using an unsupervised hierarchical clustering strategy. A module consisting of 18 mRNAs and one lncRNA (k-clique=12) (Figure 3A) separated the 106 samples into two major groups (32 vs. 74 patients; a third group that included 3 samples was removed to generate the survival curve due to the limited number of samples) based on the expression patterns of the 19 molecules (Figure 3B). Survival analysis showed a difference in disease-free survival (DFS) between these two patient subgroups (log-rank test p=2.8e-02; Figure 3C), indicating the module's prognostic potential, and the potential for each molecule to act as a candidate biomarker in the prediction of LC clinical outcomes. Notably, the only lncRNA in the module, H19, was an inflammatory hub lncRNA in the above analysis, and H19 alterations are associated with metastasis in lung, colorectal and bladder cancers, and multiple myeloma [21–23].

### The 18-mRNA and one-lncRNA module in LC clinical outcome assessment

**Figure 4:**
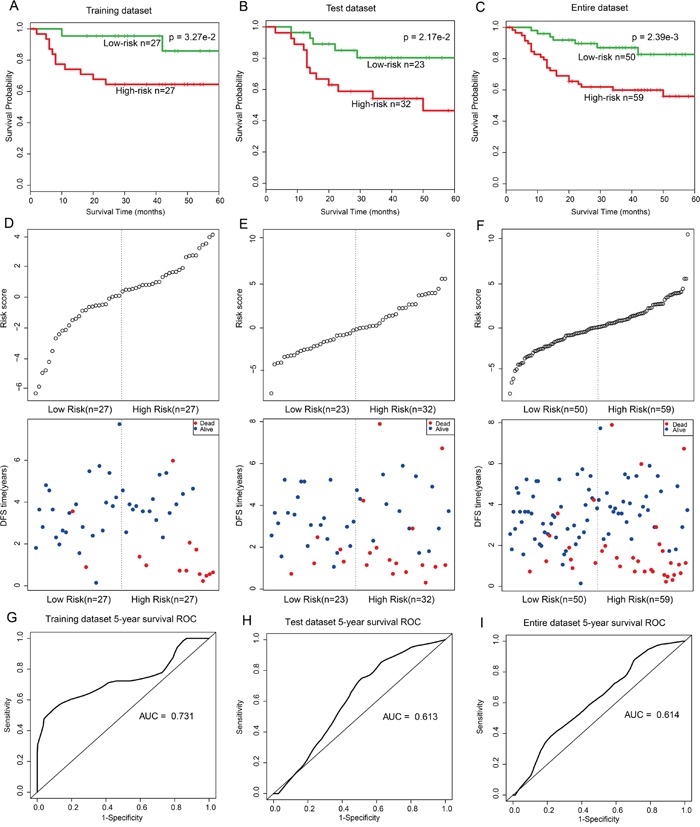
Survival curves and risk score analysis of 18-mRNA and one-lncRNA module in training, test, and entire datasets, respectively **A-C**. KM survival curves for disease free survival of training, test and entire datasets patients respectively with high and low risk scores. P value was calculated using the log-rank test. **D-F**. Risk score analysis of the module in training, test and entire datasets patients respectively. **G-I**. Receiver operating characteristic (ROC) curve analysis and area under the curve (AUC) value of the ROC curve indicating the sensitivity and specificity of the module for survival prediction.

To further validate the prognostic performance of the 18-gene and one-lncRNA module, the module was fitted in a multivariate Cox regression model with DFS as the dependent variable and other clinical information as covariables. A risk score model of molecules in this module was constructed according to a linear combination of expression values weighted by the regression coefficients derived from multivariate Cox regression analysis (Cox regression coefficients are listed in Supplementary Table 4). Molecules with larger Cox regression coefficient absolute values, such as SNTB2, have greater potentials to influence patient mortality risk. All patients were randomly divided into two subsets: training (n=54) and test (n=55). In the training dataset, patients were divided into high- (n=27) and low-risk groups (n=27) using the median risk score (-0.09) as the cut-off. Patients in the high-risk group had shorter survival times than those in the low-risk group (log-rank p=3.27e-2, Figure 4A). This module was further validated in the independent test dataset (log-rank p=2.17e-2, Figure 4B) and the entire dataset (log-rank p=2.39e-3, Figure 4C).

After further adjusting for age and grade, univariate analysis indicated that this module, as an independent risk factor, was associated with LC patient overall survival in the training (Hazard Ratio (HR)=2.72, 95% CI: 1.68–4.39, p=4.47e-5), test (HR=3.15, 95% CI: 1.12–8.85, p=2.93e-2), and whole (HR=1.20, 95% CI: 1.09–1.33, p=4.08e-4) datasets (Table 1). The distribution of gene risk scores and the survival statuses of the three datasets are shown in Figure 4D–4F. Patients with high-risk scores tended to present poorer clinical outcomes compared to patients with low-risk scores. A time-dependent receiver operating characteristic (ROC) curve analysis was performed to evaluate sensitivity and specificity for module survival prediction in the entire GEO dataset. The module achieved area under the curve (AUC) values of 0.731, 0.613, and 0.614 in the training, test, and entire datasets respectively, revealing a superior prediction performance (Figure 4G–4I).

**Table 1:** Univariate and multivariate Cox regression analysis of the 18-mRNA and one-lncRNA module in LC patients

Variables	Univariate analysis			Multivariate analysis		
	HR	95% CI of HR	P-value	HR	95% CI of HR	P-value
Training dataset						
18-mRNA and one-lncRNA module	2.72	1.68-4.39	<0.0001	2.83	1.65-4.84	<0.0001
Grade	1.27	0.590-2.71	0.546	0.837	0.319-2.20	0.719
Age	0.992	0.938-1.05	0.793	1.02	0.941-1.10	0.666
Test dataset						
18-mRNA and one-lncRNA module	3.15	1.12-8.85	2.93e-2	3.34	1.18-9.43	2.30e-2
Grade	0.824	0.426-1.59	0.565	0.731	0.385-1.39	0.336
Age	1.04	1.00-1.09	5.20e-2	1.04	0.997-1.09	6.60e-2
Entire dataset						
18-mRNA and one-lncRNA module	1.20	1.09-1.33	<0.0001	1.20	1.08-1.33	<0.0001
Grade	1.00	0.608-1.65	0.999	0.902	0.541-1.51	0.694
Age	1.02	0.989-1.06	0.192	1.02	0.985-1.06	0.249

**Figure 5:**
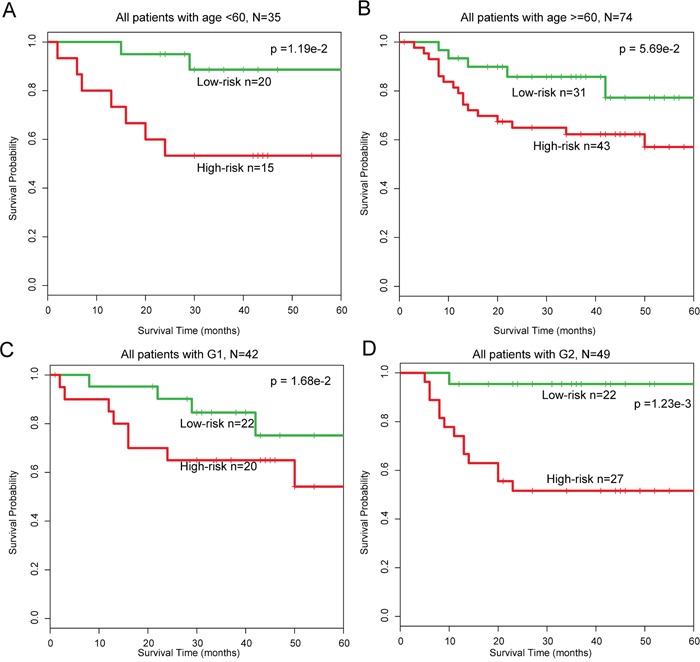
Stratification analysis of all patients based on age and grade **A-B**. KM survival curves of patients stratified by age based on the 18-mRNA and one-lncRNA module (age < 60, N = 35; age >= 60, N = 74). **C-D**. KM survival curves of patients stratified by grade based on the 18-mRNA and one-lncRNA module (G1, N = 42; G2, N = 49).

In addition, multivariate analysis was performed to investigate the independence of the module with respect to the other clinical factors. The results demonstrated that designation of high- and low-risk groups remained statistically significantly independent of other clinical factors in training (HR=2.83, 95% CI: 1.65–4.84, p=1.56e-4), test (HR=3.34, 95% CI: 1.18–9.43, p=2.30e-2), and entire (HR=1.20, 95% CI: 1.08–1.33, p=4.22e-4) datasets (Table 1). Data stratification analysis results in GSE27020 indicated that the module was age and grade independent, as it performed equally well in the two patient age groups (log-rank test p=5.70e-2 for patients >60 years of age, and p=1.10e-2 for patients <60 years of age) and in patients with grades G1 or G2 disease (log-rank test p=1.68e-2 for G1 group and p=1.00e-3 for G2 group) (Figure 5A–5D). Taken together, our data suggest that the 18-mRNA and one-lncRNA module may be a useful prognostic indicator in LC.

## DISCUSSION

Laryngeal cancer (LC) originating from the head and neck region is the second most common upper-aerodigestive cancer [24, 25], and patient prognosis remains poor. The ceRNA hypothesis, which states that ceRNAs act as molecular sponges for miRNAs to de-repress the miRNA target genes, has been proposed as a novel post-transcriptional gene expression regulatory mechanism [26]. In this study, on the basis of the ceRNA hypothesis and the fact that inflammation likely promotes LC, we used LC patient lncRNA and mRNA expression profiles combined with experimentally validated miRNA-target interactions to construct an inflammatory gene-associated ceRNA network (IceNet). This network consisted of 935 molecules (including 190 inflammatory genes, 10 lncRNAs and 735 mRNAs) and 5,415 interactions. IceNet is a novel method for exploring the roles of inflammatory gene-associated ceRNA networks in LC initiation and progression. To investigate the IceNet structure and the function of its components, we analyzed the topological properties of the inflammatory genes and their ceRNAs within the network. We found that inflammatory genes had lower degrees and CCs than their ceRNAs, indicating that these ceRNAs were central to IceNet. BCs of hub inflammatory gene ceRNAs, on the other hand, were higher than those of the inflammatory genes themselves, indicating that these ceRNAs played critical communication roles with molecules in IceNet.

Furthermore, we identified a potential prognostic module within IceNet that included 18 mRNAs and one lncRNA. The module identified high- vs. low-risk patients independent of clinical factors, such as age and grade. The potential targets of this module, i.e. the first neighbors of the 18-mRNA and one-lncRNA, were associated with different types of cancers, including LC. For example, AMOT appears to regulate breast cancer cell proliferation and invasion [27]. By regulating the miR-107/CDK6 interaction, the lncRNA NEAT1 may promote laryngeal squamous cell cancer [28]. Encoded by SPAM1, PH-20 was upregulated in primary LC tissues, and may be an LC prognostic marker [29].

Additionally, several of the 19 molecules in the module themselves are reportedly associated with various cancers. The lncRNA H19 may promote glioma development and invasion [12]. A balance of acetylation and deaceylation by ATAT1/HDAC6 enzymes regulates breast cancer cell migration and invasion [30]. BMP8B mediated pancreatic cancer cell survival and regulated pancreatic cancer progression [31]. CRY2 may promote breast cancer aggressiveness, possibly via epigenetic modifications [32], and its degradation promotes chemoresistance in colorectal cancer [33]. LIMK1 and LIMK2 are important for pancreatic cancer cell metastasis and tumor cell-induced angiogenesis [34].

In conclusion, the 18-mRNA and one-lncRNA module identified in our study provides a novel mechanism for potentially improving LC patient prognostic predictions. Applying the module clinically to differentiate high- and low-risk patients could inform therapeutic decision-making and ultimately improve patient outcomes. In addition, our identification of potential LC prognostic biomarkers based on an inflammatory gene-related, ceRNA network analysis demonstrated the potential importance of hub ceRNAs in disease development and progression.

## MATERIALS AND METHODS

### Data collection

Laryngeal cancer patient datasets, corresponding clinical information, and mRNA expression profiles were collected from the publicly available GEO database. After removing patients without disease-free survival (DFS) information, a total of 109 patients were selected from the GSE27020 [35] dataset on the Affymetrix HG-U133A platform.

lncRNA levels from the Affymetrix-based patient expression profiles were obtained by repurposing the microarray probes using a previously described method [36]. First, Affymetrix HG-U133A probesets from the Affymetrix website (http://www.affymetrix.com) were re-mapped to the human genome (GRCh38/hg38) using SeqMap33 with no mismatch [37]. Second, the chromosomal positions of those probes, which were uniquely mapped to the human genome, were matched to the chromosomal positions of lncRNAs derived from GENCODE (release 21, GRCh38) [38]. A total of 909 probes (or probe sets) and 649 corresponding lncRNA genes were obtained (Supplementary Table 5). Multiple probes (or probe sets) that mapped to the same lncRNA were combined using the median expression value of the probes (or probe sets).

Human miRNAs and targets genes were collected from miRTarBase (version 6.1) [39], which provides high quality experimentally validated miRNA-target interaction relationships manually curated from published experiments. A total of 322,388 non-redundant miRNA-target interactions were used in our study. The experimentally validated miRNA-lncRNA interactions were downloaded from starBase v2.0 [40], including 10,212 miRNA-lncRNA interactions.

A list of Gene Ontology (GO) [41] terms related to the inflammatory response were obtained from a previous study [42]. The genes annotated to these inflammatory-associated GO terms were obtained from the AmiGO2 tool [43] of the Gene Ontology Consortium.

### Construction of inflammatory gene-related ceRNA network

Using the experimentally supported miRNA-mRNA and miRNA-lncRNA interaction data, we primarily followed the two principles listed below to identify inflammatory gene-related ceRNAs (IceRNAs, including mRNA-mRNA and mRNA-lncRNA pairs) in LC: trans-regulatory ceRNA crosstalk increased with high miRNA regulatory similarity and ceRNA pair co-expression. First, a hypergeometric test was executed for each possible ceRNA pair separately. For each given ceRNA pair A and B, we identified miRNAs that regulated them both (A ∩ B). Then, the probability for A and B was calculated according to

$$P=1-F\left(x|N,K,M\right)=1-\sum_{t=0}^{x}\frac{\left(\begin{array}{c} K\\ t \end{array}\right)\left(\begin{array}{c} N-K\\ M-t \end{array}\right)}{\left(\begin{array}{c} N\\ M \end{array}\right)}$$ (1)

where N was the number of all miRNAs, K and M were the total number of miRNAs regulated by A and B respectively, and x was the number of miRNAs shared between the pairs. Only pairs regulated by at least one mutual miRNA were analyzed in our study. Candidate ceRNA pairs with P-values <0.05 were used for the subsequent analysis. Second, to further identify co-expressed ceRNA pairs, Pearson correlation coefficients were calculated based on expression of the pairs:

$$\rho_{X,Y}=\frac{\text{cov}(X,Y)}{\sigma_{X}\sigma_{Y}}$$ (2)

where cov (X, Y) is the covariance of variables X and Y, and σ_X_ and σ_Y_ are the standard deviations for X and Y, respectively. To reduce false positive rates, only ceRNA pairs with Pearson correlation coefficient >0.5 were included in further analyses. This threshold was used in a previous study to identify functional activated (competing) ceRNA networks across 12 cancers [44].

Based on the two criteria above, a well-correlated ceRNA network was constructed. The first neighbors of inflammatory genes in the ceRNA network were then selected as a subnetwork using Cytoscape 3.2.0 [45]. First neighbors were defined as the nodes directly interacting with inflammatory genes. Then, the IceNet was built by extracting the maximal connected components of the subnetwork.

### Network and functional enrichment analysis

Topological properties, including degree, betweenness centrality (BC), and closeness centrality (CC), were used to decipher the structure of IceNet and to identify “important” molecules. Degree was used to determine the number of neighbors for each node. BC represented the key role of a node in communication and information diffusion [46]. Node CC measured local cohesiveness, i.e. how close a node is to other nodes. IceNet visualization and topological properties were analyzed using Cytoscape 3.2.0 [45] and the R package, igraph. Module detection was accomplished using the cFinder algorithm [47], and identification and visualization of overlapping dense groups of nodes was done using the Clique Percolation Method (CPM) [48].

Genes were functionally annotated to identify enriched pathways based on DAVID Bioinformatics Resources (http://david.abcc.ncifcrf.gov/, version 6.7) [49]. Functional categories were visualized and clustered using the EnrichmentMap plugin in Cytoscape 3.2.0 [50]. Nodes and distances between nodes were automatically generated to place higher similarity gene-sets closer together. These clusters and their biological functions could be easily identified manually.

### Survival analysis

A risk score was calculated based on expression of each molecule in IceNet weighted by its prognostic power from a multivariate Cox regression analysis:

$$\text{RiskScore}=\sum_{i=1}^{n}r_{i}\text{Exp}(i)$$ (3)

where n is the number of prognostic components, Exp(i) is the expression of prognostic component I, and ri is the estimated regression coefficient of component i in the multivariate Cox regression analysis. The survival predictive ability of the module was then assessed based on its ability to separate patients into two subgroups by the mean risk score value. The Kaplan-Meier (KM) method was used to estimate the survival curves between high- and low-risk groups. Statistical significance was evaluated using the two-sided log-rank test. In addition, we evaluated the sensitivity and specificity of the module for survival prediction using receiver operating characteristic (ROC) curve analysis and the area under the ROC curve (AUC).

## SUPPLEMENTARY TABLES








