# Supplementary material for: Identification of laryngeal cancer prognostic biomarkers using an inflammatory gene-related, competitive endogenous RNA network
Source: Oncotarget. 2016 Nov 25;8(6):9525–34. doi: 10.18632/oncotarget.13627 (PMC5354750; doi:10.18632/oncotarget.13627)
Supplement: Supplementary file 1 [file oncotarget-08-9525-s001.pdf]

Identification of laryngeal cancer prognostic biomarkers using an inflammatory gene-related, competitive endogenous RNA network

SUPPLEMENTARY TABLES

Supplementary Table 1: Interactions in IceNet

See Supplementary File 1

Supplementary Table 2: Enriched GO-FAT BP and KEGG pathways of mRNAs in IceNet

See Supplementary File 2

Supplementary Table 3: Gene list of 9 modules identified by cFinder

K-clique=13	"A1CF, AAK1, AGFG2, ATAT1, BMP8B, C16orf70, ENSG00000130600, FZD9, LIMK1, SEMA7A, SLC28A1, SNTB2, SPIB, VPS53"
K-clique=12-1	"ABCC6, ASGR2, CBX2, ENSG00000267858, FZD9, HOXC8, IRF4, MTHFR, NCOR2, NR6A1, RS1, SNTB2, TTLL5"
K-clique=12-2	"A1CF, AAK1, AGFG2, ATAT1, BMP8B, C16orf70, CCDC30, CRY2, ENSG00000130600, FZD9, LIMK1, SEMA7A, SLC28A1, SLC6A4, SMAD6, SNTB2, SP4, SPIB, VPS53"
K-clique=12-3	"ABCC6, CA8, CBX2, ENSG00000267858, FZD9, HOXC8, IRF4, MTHFR, NR6A1, RARB, SNTB2, STK38, TTLL5"
K-clique=11-1	"COL10A1, COL3A1, COL4A1, COL5A2, FBN1, LEPRE1, LIMS1, MMP2, PDGFRB, SERPINH1, SPARC"
K-clique=11-2	"ABCC6, ADRB3, ASH1L, B3GALT5, BSN, C6, CA8, GREB1L, GRIK3, GRIN2A, IL5RA, LONRF3, MC2R, MOBP, PRRG3"
K-clique=11-3	"AQP6, B3GALT5, BTNL3, CAMK2A, DISC1, GP5, IQSEC3, KCNA5, LLGL1, MAT1A, NCKIPSD, NCOR2, NUDT7, PKNOX2, SCUBE3, SIT1, STK38, THRA, TTC21B, ZKSCAN3"
K-clique=11-4	"A1CF, AAK1, AGFG2, ATAT1, BMP8B, C16orf70, CCDC30, CRY2, ENSG00000130600, FZD9, KCND3, LIMK1, LUZP2, PARD6B, SEMA7A, SLC28A1, SLC6A4, SLCO5A1, SMAD6, SNTB2, SP4, SPIB, VPS53"
K-clique=11-5	"ABCC6, AMOT, ASGR2, CA8, CBX2, CDK6, ENSG00000267858, FZD9, HOXC8, IRF4, MTHFR, NCOR2, NR6A1, RARB, RS1, SNTB2, STK38, TTLL5"

Supplementary Table 4: The Cox regression coefficients of the 18 mRNAs and a lncRNA module

Gene	M2coef
A1CF	1.269563246
AAK1	0.372164346
AGFG2	−1.135966615
ATAT1	0.770906898
BMP8B	0.363695
C16orf70	−0.846315149
CCDC30	−1.030200887
CRY2	0.778204734
H19	−1.350619899
FZD9	0.234983252
LIMK1	−0.189328236
SEMA7A	0.98547374
SLC28A1	1.609487473
SLC6A4	−0.529571298
SMAD6	0.75485728
SNTB2	−2.490323076
SP4	0.991397178
SPIB	−0.685257841
VPS53	−0.694578524

Supplementary Table 5: The detailed information of probe sets and their corresponding lncRNAs in the Affymetrix HG-U133A platform used in our study

See Supplementary File 3
